# An Emergency Medical Services Toolkit for Improving Systems of Care for Stroke in North Carolina

**Published:** 2009-03-15

**Authors:** Ishmael Williams, Greg Mears, Jenny Wilson, Cindy Raisor

**Affiliations:** Epidemiology and Surveillance Branch, Division for Heart Disease and Stroke Prevention, National Center for Chronic Disease Prevention and Health Promotion, Centers for Disease Control and Prevention; University of North Carolina at Chapel Hill, Chapel Hill, North Carolina; University of North Carolina at Chapel Hill, Chapel Hill, North Carolina; EMS Performance LLC, Chapel Hill, North Carolina

## Abstract

The Centers for Disease Control and Prevention is partnering with the National Association of Chronic Disease Directors and the North Carolina Office of EMS to design, develop, and implement an emergency medical services (EMS) performance improvement toolkit to evaluate opportunities to improve the emergency identification and treatment of acute stroke. The EMS Acute Stroke Care Toolkit is being developed, tested, and implemented in all 100 counties in the state by the EMS Performance Improvement Center, the agency that provides technical assistance for EMS in North Carolina. The toolkit helps each EMS system in defining, measuring, and analyzing their system of care and promotes collaboration through public education, regional stroke planning with hospitals, EMS service configuration, EMS staffing patterns, EMS education, and timely care delivery. We outline the issues surrounding acute stroke care, the role of emergency medical systems in stroke care, and the components of the EMS Acute Stroke Care Toolkit designed to improve EMS systems and outcomes for stroke patients.

## Background

Stroke is the third leading cause of death in the United States. Each year, approximately 700,000 people suffer a first-time or recurrent stroke, approximately 25% of whom die from stroke-related causes, and another 15% to 30% of whom remain permanently disabled (1). The Centers for Disease Control and Prevention (CDC) reported that in 1999, approximately 48% of stroke deaths occurred pretransport (before transport to a hospital emergency department) (2). The percentage of pretransport deaths by state ranged from 23% to 67%, and 8 states had proportions greater than 60%. The study found that ischemic strokes, those strokes caused by a blockage in an artery that supplies blood to the brain, accounted for 68% of all out-of-hospital stroke deaths (2). A follow-up study by CDC found that, although the stroke death rate decreased from 61.6 per 100,000 in 1999 to 56.2 per 100,000 in 2002, the percentage of out-of-hospital deaths was unchanged. In 2002, of 162,672 deaths from stroke, 49% of the patients died before being transported to a hospital (3).

This high rate of death due to stroke before arrival at the hospital is troubling, considering the promising stroke treatment options that exist. One treatment for ischemic stroke, thrombolytic (“clot-busting”) therapy, was approved for use in the United States by the Food and Drug Administration in 1996 and works well if administered within the first 3 hours of the onset of symptoms. However, in 2004, only 3% to 8.5% of stroke victims received this treatment (4). Reasons most frequently cited for not receiving treatment were delays in 1) calling 9-1-1, 2) transporting patients to a hospital capable of handling stroke patients, and 3) diagnosing and treating patients after they arrive at the hospital (4).

Retrospective studies have found that the biggest portion of the delay between onset of symptoms and emergency treatment is the time it takes for a patient to recognize the signs of stroke and decide to seek medical care (5-8). Between one-half and three-quarters of ischemic stroke patients do not arrive at the hospital within the 3-hour window of treatment that is needed to make an assessment and begin therapy. Some of the factors in the delays include lack of knowledge regarding 1) stroke symptoms, 2) treatment options, and 3) the need for quick therapy (5-8).

Delays in treating stroke also occur because of poor recognition of stroke by 9-1-1 dispatchers and misdiagnosis of stroke by emergency medical services (EMS) personnel. Dispatch is a crucial link in the chain of care, yet dispatchers miss as many as 70% of stroke cases because they do not have the understanding or tools to properly assess the symptoms reported by callers. A similarly high misdiagnosis rate (61%) was documented for the responding EMS personnel when diagnosing stroke in the field (9).

These findings underscore the challenges facing emergency systems of care. The lack of close coordination of stroke care among health care providers has resulted in a fragmented system for stroke prevention, emergency care, treatment, and rehabilitation. Several key stroke care stakeholders have made recommendations to address these problems (10-19). Their recommendations call for better integration of the facilities, agencies, and professionals that provide stroke care. These recommendations include rapid access to EMS, use of diagnostic algorithms and EMS protocols that reflect the most current stroke treatment recommendations and dispatch EMS with the most rapid emergency response possible, direct involvement of emergency physicians and stroke experts in designing protocols and training, stroke assessment and thrombolytic screening, and rapid transport to a stroke center (16).

To address the recommendations from these organizations, CDC, the National Association of Chronic Disease Directors, and the North Carolina Office of EMS are developing and implementing an EMS performance improvement toolkit (a statistical analysis report on EMS patient data) to evaluate opportunities for improvement in the emergency identification and treatment of acute stroke. This toolkit is being implemented by using an evidence-based approach established by the National EMS Information System (NEMSIS) to define, document, and evaluate EMS service delivery.

## National EMS Information System

NEMSIS is a standardized data set and data transmission standard for service delivery and patient care. It has been endorsed by all 56 states and territories and has been implemented in 42 states (20). NEMSIS was initially created through an industry-wide consensus process. The goal was to establish an electronic EMS data system at the local level that would collect and maintain EMS service delivery and patient care data. These data represent an EMS patient care event beginning with the 9-1-1 call to activate EMS through the transport or disposition of the patient by EMS. A subset of these data is electronically submitted to a state EMS database. The state data are used to support the state EMS infrastructure and to drive policy, funding, and other EMS needs. A subset of the state EMS database is submitted to a national EMS database. This national database describes the EMS industry, service delivery, and patient care from a national perspective. Data from the national database are used to support EMS policy, advocacy, funding, and education (21). Twelve states, including North Carolina, provide data for the national EMS database as of January 2009 (20).

NEMSIS uses a standardized data dictionary, which permits data-driven analyses and assessment of an EMS system's performance, procedures, personnel, and patient outcomes. Standardized data also enable comparison or benchmarking across jurisdictional and state boundaries and describe national trends. As trends and performance benchmarks are identified, we can identify and define EMS needs, develop evidence-based EMS treatment protocols, support and justify EMS funding, establish EMS policy, and target EMS research.

EMS toolkits are designed to improve the quality of EMS care by providing tools to measure and analyze key processes or components of using a "systems of care" approach. By defining, measuring, analyzing, and recommending adjustments to the key processes associated with EMS service delivery, personnel performance, and patient care, an EMS system can make changes to optimize performance over time. Toolkits identify best practices that can be shared anonymously to allow comparisons and benchmarking of service delivery and patient care among similar EMS systems across the state.

The EMS toolkits are one component of a statewide program for EMS quality improvement using the data-driven quality control strategy Six Sigma. First used in manufacturing by Motorola, Six Sigma has been tested and adapted to areas including health care delivery. It promotes incremental quality improvement through processes for defining, measuring, analyzing, improving, and controlling performance (22).

## The North Carolina EMS Acute Stroke Care Toolkit

The North Carolina Office of EMS created the NEMSIS-based North Carolina Prehospital Medical Information System (PreMIS), which is maintained by the EMS Performance Improvement Center (EMSPIC) at the University of North Carolina at Chapel Hill. All 100 North Carolina counties are required by law to provide data to the North Carolina Office of EMS through the PreMIS data system. More than 1 million EMS calls each year occur within North Carolina and are recorded in the database.

A focal point of EMSPIC has been to provide ongoing technical support to local EMS systems by developing and implementing EMS performance improvement toolkits. These toolkits are data analysis programs that enable statistical analysis of an EMS system's care for a specific patient population. The toolkits use SAS software (SAS Institute, Cary, North Carolina) and create graphic reports on a Web browse-based interface accessible by authorized EMS managers. The toolkits help local and state EMS systems evaluate and improve EMS service delivery, personnel performance, and patient care.

Development of toolkits for EMS systems was initiated in 2004 under a 3-year grant provided by The Duke Endowment. Five EMS system toolkits were initially developed and implemented; they addressed system response time, acute trauma care, cardiac arrest care, acute pediatric care, and acute cardiac care. The EMS Acute Stroke Care Toolkit, funded by CDC, was an additional EMS toolkit developed and implemented by EMSPIC in 2007 (22).

The design of the EMS Acute Stroke Care Toolkit included industry standards when possible. If there were no accepted industry standards, a consensus process was used. Two expert panels convened to identify and agree on emergency stroke care indicators and to convert the indicators into performance measures and a basic design for the toolkit. The panel members represented a broad spectrum of stroke and EMS expertise (23).

The EMS Acute Stroke Care Toolkit reports on many broad indicators of systems of care for stroke, including stroke incidence and death rates from CDC, demographic characteristics of stroke patients, and the socioeconomic background of the service area of the EMS system from US Census Bureau statistics. The toolkit tracks EMS involvement in local community education and stroke prevention programs and EMS participation in the North Carolina Stroke Initiative — a statewide effort to engage EMS systems and hospitals to optimize care for stroke patients focusing on critical time intervals and interventions. To promote cardiovascular health in the EMS workforce, the toolkit recommends implementing an EMS workforce health and safety plan in each EMS system (24).

The EMS Acute Stroke Care Toolkit uses approximately 60 data elements to describe and measure the performance and effectiveness of an EMS system, beginning with the initial 9-1-1 call through the treatment and disposition of the stroke patient. Data collected during an EMS event is closely monitored by the EMSPIC staff for accuracy and completeness at the local level. This monitoring ensures decisions are made with the best possible information. Data quality, completeness, and validity are analyzed and reported as a component of each toolkit.

From the perspective of patient outcome, the EMS Acute Stroke Care Toolkit focuses on 5 key patient care interventions:

- Prompt recognition of stroke through the use of stroke screening.
- Documentation of stroke symptom onset.
- Screening the blood glucose of the patient for hypoglycemia.
- Maintaining EMS scene times of 10 minutes or less.
- Rapid transport (with early notification) to a stroke center.

An extensive set of observations is collected for each EMS event. These data are used to measure and analyze the EMS service delivery and stroke care interventions. The observations include time intervals for each stage of response, treatment, and transport (Table 1). The time fractions collected can be divided as follows: 1) the time to dispatch an ambulance after the 9-1-1 call, 2) the time to get the ambulance moving after dispatch, 3) the time to get to the scene, 4) time spent at the scene, and 5) the time to get the patient to the hospital after leaving the scene.

The EMS Acute Stroke Care Toolkit calculates (within a user-specified range of dates) EMS system time intervals for all EMS events in the database. These intervals present in hours, minutes, and seconds the minimum, maximum, and average time to respond to a call; the 90% fractile (the maximum response time for 90% of events); and the standard deviation. Each of these calculations are made with 5 levels of comparison: 1) the EMS system’s emergency calls (lights and sirens used), 2) the EMS system’s stroke events (stroke patients), 3) the entire state’s stroke events, 4) stroke events for EMS systems in the state that serve similar population sizes, and 5) stroke events for EMS systems in the state that serve similarly sized areas (Figure) (24).

**Table:** 

System	Events (n)	Minimum Value	Maximum Value	Average Value	90% Fractile	Standard Deviation
EMS system (all emergent)	593	0:00:00	0:29:00	0:06:28	0:12:00	0:04:19
EMS system (acute stroke)	2	0:00:00	0:06:00	0:02:20	0:06:00	0:03:13
State	41,430	0:00:00	1:35:00	0:06:17	0:11:00	0:04:09
Similar EMS system (by population)	15,073	0:00:00	1:09:00	0:06:07	0:11:00	0:04:00
Similar EMS system (by area)	16,182	0:00:00	1:35:00	0:06:19	0:12:00	0:04:32

**Figure.**
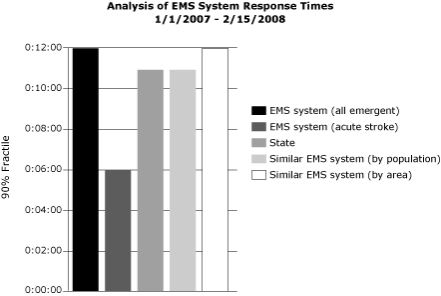
An example analysis of an emergency medical services (EMS) system's response time in hours, minutes, and seconds (time beginning with the dispatch of an EMS vehicle and ending with the arrival of the EMS vehicle at the scene of an EMS event) for January 1, 2007, through February 15, 2008. Only emergent events when lights and sirens were used are included in the calculations. All of the EMS system's events are compared with acute stroke events, the entire state's emergent events, and the response times for stroke events for similar EMS agencies (by population and by area).

Uncontrollable factors that affect the reported time intervals by causing delays are weather, traffic or crowds, safety concerns, vehicle failure, vehicle crash, language barriers, distance, problems with directions, and hazardous materials. Such factors are reported for the acute stroke calls only for response, scene, and transport. The toolkit reports the total number of delays by type and the percent of total EMS responses each delay type represents.

EMS treatment protocols for stroke care are reported in depth, and protocol compliance is tracked at the individual EMS professional and at the stroke patient level (Table 2). North Carolina's EMS protocols are a set of best practices determined by the North Carolina College of Emergency Physicians (25) and other stroke experts. The stroke protocol requires documentation of onset time of symptoms, completion of stroke screen, blood glucose (for hypoglycemia) and thrombolytic screening (to identify patients who would benefit from clot-busting drug treatment), an EMS scene time of 10 minutes or less, and documentation of the patient's cardiac rhythm for to check for arrhythmias.

The EMS Acute Stroke Care Toolkit summarizes all of the EMS data elements stored in the PreMIS database for a period of time determined by the user. The EMS system's administrator compares his or her system's results for a selected time period with the state's results and results from other EMS systems that cover similarly sized areas and populations. The toolkit also allows EMS administrators to make historic comparisons to evaluate the effects of any change or intervention. The EMS Acute Stroke Care Toolkit has 28 interventions or recommendations for improvement (Table 3). Each of these interventions is specific to a performance measure or finding in the toolkit. Each time a toolkit report is generated, the EMS system receives 12 to 15 recommendations or suggestions for improvement.

Toolkit-based interventions also serve as a built-in evaluation for any EMS system changes implemented to improve EMS service delivery, personnel performance, or patient care. Once an intervention has been implemented in an EMS system, all data collected will reflect this change. After enough data on EMS events or patient records have been collected, the toolkit can be regenerated to assess the performance improvement since the previous toolkit report and the progress achieved in meeting the benchmarks.

## Conclusions

The EMS Acute Stroke Care Toolkit is a component of a larger nationwide movement to improve stroke outcomes by integrating and improving the entire chain of medical care for stroke from the recognition of signs and symptoms to recovery and rehabilitation. The toolkit addresses the first level of emergency care — response time, diagnosis, collection of medical history and symptom onset times, stabilization, thrombolytic screening, and quick transport to a hospital capable of handling stroke care. All these elements are critical if stroke patients are to obtain the best chance for positive outcomes from definitive treatment. The EMS Acute Stroke Care Toolkit and NEMSIS represent a major step in identifying the evidence-based EMS practices needed to improve emergency stroke treatment. Each toolkit identifies interventions to improve EMS, gauges the success of the interventions, and helps identify other areas that need incremental improvement.

The next phase of the EMS Acute Stroke Care Toolkit, which has been funded for 2 years starting in 2009, will include a toolkit usability study that will gather input from EMS managers, evaluate the effectiveness of the toolkit for incremental improvement, and implement the toolkit in South Carolina and West Virginia, which have recently started using PreMIS. CDC is also working in North Carolina with the EMSPIC and the Coverdell Stroke Registry for hospitals to link EMS and hospital data sets so that patient outcomes can be assessed across the spectrum of care. This effort involves linking the patient records in the 2 databases and evaluating EMS protocols with respect to emergency care and hospital care. Plans also include a version of the toolkit that could be used by other states and local EMS systems that use the NEMSIS standard.

An EMS Acute Stroke Care Toolkit with real data (blinded to patient and EMS system identity) is available at www.emspic.org/?q=node/27. Additional information on this and other EMS toolkit projects and the EMSPIC is available at www.emspic.org.

## Figures and Tables

**Table 1:** Key Emergency Medical Services (EMS) Time Intervals Used Within an EMS System to Evaluate EMS Service Delivery and Acute EMS Stroke Care, North Carolina, 2007

**Time Element**	Description
Dispatch center	Beginning with the call to 9-1-1 and ending with the dispatch of an EMS vehicle to the scene
"Wheels rolling"	Beginning with the dispatch of the EMS vehicle and ending with the movement of the vehicle to the scene
EMS response	Beginning with the movement of the EMS vehicle toward the scene and ending with the arrival of the EMS vehicle at the scene
Scene	Beginning with the arrival of the EMS vehicle at the scene and ending with the EMS vehicle leaving the scene with the patient en route to the hospital
Transport	Beginning with the EMS vehicle leaving the scene with a patient en route to a hospital and ending with the EMS vehicle arriving at the destination hospital
Total patient contact	Beginning with the call to 9-1-1 and ending with the EMS vehicle arriving at the destination hospital

**Table 2:** Emergency Medical Services (EMS) Acute Stroke Care Toolkit Protocol Performance Measures^a^, North Carolina, 2007

**Protocol**	Description
Symptom onset time noted	Time of onset in hours and minutes
Stroke screen information obtained	Cincinnati Stroke Screen or Los Angeles Prehospital Stroke Screen used
Glucose checked	Blood glucose check for hypoglycemia
Thrombolytic screen	A checklist of contraindications to thrombolytic therapy
Scene time ≤10 min	Total time spent at scene of stroke
Cardiac rhythm	Cardiac rhythm checked for arrhythmias

a These measures are analyzed at 3 levels: 1) each stroke patient is analyzed to determine whether all performance measures were met, 2) each EMS professional's care is analyzed to determine how many stroke patients received all of the performance measures, and 3) the EMS system is analyzed to determine how many stroke patients in the EMS system received all of the performance measures.

**Table 3:** Intervention List for Improving an Emergency Medical Services (EMS) Acute Stroke Care Toolkit, North Carolina, 2007^a^

**Intervention**	**Description of Problem**
Patient identification data quality	Missing data elements that prevent the identification of stroke patients
Stroke care (missing patient care record data) data quality	Missing data elements important to the evaluation of stroke care
Stroke care (incomplete patient care record data) data quality	Underreporting of patient care report data elements important to the evaluation of stroke care
Stroke care (missing additional data) data quality	Missing additional data elements important to the evaluation of stoke care
Protocol use	Incomplete protocol information provided
Reason for encounter	Incomplete reason for encounter documentation provided
First responder coverage	Less than 100% first-responder coverage
Dispatch center stroke recognition training	Less than 100% of EMS dispatchers trained in stroke recognition
EMS personnel trained in stroke recognition	Less than 100% of EMS personnel trained in stroke recognition
Emergency medical dispatch	The dispatch center is not an emergency medical dispatch center
Wireless 9-1-1	The dispatch center does not have wireless 9-1-1 capabilities
Dispatch center time	Prolonged EMS system dispatch center times noted
EMS "wheels rolling" time	Prolonged EMS system "wheels rolling" times noted
EMS response time	Prolonged EMS system response times noted
EMS scene time	Prolonged EMS system scene times noted
EMS response delays	EMS system response delays noted
EMS scene delays	EMS system scene delays noted
EMS transport delays	EMS system transport delays noted
EMS personnel data quality scores	Increased (poor) EMS personnel documentation data quality scores noted
EMS system data quality scores	Increased (poor) EMS system documentation data quality scores noted
Stroke screen documentation	Incomplete or missing stroke screen documentation
Glucose level documentation	Missing blood glucose level documentation
Reperfusion checklist documentation	Missing documentation of a thrombolytic screen or reperfusion checklist
Duration of symptoms	Missing or incomplete duration of symptoms provided
Cardiac rhythm documentation	Missing documentation of cardiac rhythm
Stroke patient outcome	Suggestions for improvement of stroke patient outcome
Written stroke plan	The EMS system does not have a written stroke triage or destination plan
Hospital early notification	Missing plan to prealert hospitals of an acute stroke patient's arrival

a These interventions represent suggestions or opportunities for improvement for an EMS system. Recommendations to each EMS system are based on its specific results. An average EMS system will receive 12 to 15 interventions each time a toolkit is generated.

## References

[1] (2007). American Heart Association. Heart and stroke statistics — 2007 update at-a-glance.

[2] Centers for Disease Control and Prevention (2002). State-specific mortality from stroke and distribution of place of death — United States, 1999. MMWR Morb Mortal Wkly Rep.

[3] Centers for Disease Control and Prevention (2006). Place of death after stroke — United States, 1999-2002. MMWR Morb Mortal Wkly Rep.

[4] Reeves MJ, Arora S, Broderick JP, Frankel M, Heinrich JP, Hickenbottom S (2005). Acute stroke care in the US: results from four pilot prototypes of the Paul Coverdell National Acute Stroke Registry. Stroke.

[5] Smith MA, Doliszny KM, Shahar E, McGovern PG, Arnett DK, Luepker RV (1998). Delayed hospital arrival for acute stroke: the Minnesota Stroke Survey. Ann Intern Med.

[6] Rosamond WD, Gorton RA, Hinn AR, Hohenhaus SM, Morris DL (1998). Rapid response to stroke symptoms: the Delay in Accessing Stroke Healthcare (DASH) study. Acad Emerg Med.

[7] Morris DL, Rosamond W, Madden K, Schultz C, Hamilton S (2000). Prehospital and emergency department delays after acute stroke: the Genentech Stroke Presentation Survey. Stroke.

[8] Schroeder EB, Rosamond WD, Morris DL, Evenson KR, Hinn AR (2000). Determinants of use of emergency medical services in a population with stroke symptoms: the Second Delay in Accessing Stroke Healthcare (DASH II) Study. Stroke.

[9] Brice J, Murdock M, Acker III (2006). The stroke rapid response project: an interim report. Stroke Clinical Updates.

[10] Institute of Medicine (2001). Crossing the quality chasm: a new health system for the 21st century.

[11] Institute of Medicine (2006). The future of emergency care in the United States health system.

[12] Alberts MJ, Hademenos G, Latchaw RE, Jagoda A, Marler JR, Mayberg MR (2000). Recommendations for the establishment of primary stroke centers. Brain Attack Coalition. JAMA.

[13] Alberts MJ, Latchaw RE, Selman WR, Shephard T, Hadley MN, Brass LM (2005). Recommendations for comprehensive stroke centers: a consensus statement from the Brain Attack Coalition. Stroke.

[14] (2003). National Institute of Health. Improving the chain of recovery for acute stroke in your community: task force reports.

[15] Adams R, Acker J, Alberts M, Andrews L, Atkinson R, Fenelon K (2002). Recommendations for improving the quality of care through stroke centers and systems: an examination of stroke center identification options: multidisciplinary consensus recommendations from the Advisory Working Group on Stroke Center Identification Options of the American Stroke Association. Stroke.

[16] Schwamm LH, Pancioli A, Acker JE, Goldstein LB, Zorowitz RD, Shephard TJ (2005). Recommendations for the establishment of stroke systems of care: recommendations from the American Stroke Association's Task Force on the Development of Stroke Systems. Stroke.

[17] Pancioli A, Cantwell L, Crocco T, Eckstein M, Jauch E, Larrabee H (2007). Stroke journal report 9/24/2007. Communities play critical role in emergency stroke care, panel says.

[18] (1996). EMS implementation guide: agenda for the future.

[19] (2003). A public health action plan to prevent heart disease and stroke.

[20] (2005). National EMS Information System (NEMSIS) Technical Assistance Center. States and territory information.

[21] Mears G, Ornato JP, Dawson DE (2002). Emergency medical services information systems and a future EMS national database. Prehosp Emerg Care.

[22] (2007). EMS toolkits.

[23] (2006). North Carolina stroke advisory council formed: legislatively mandated council holds first meeting. [press release].

[24] EMS Performance (2004-2008). EMS Performance Improvement Center. EMS System Acute Stroke Care Toolkit Draft.

[25] Suspected stroke (2005). Suspected stroke. NCCEP protocol 30.

